# Evaluating the clinical use and safety of LumenEye in an outpatient colorectal clinic: a single-centre retrospective cohort study

**DOI:** 10.1007/s10151-025-03279-0

**Published:** 2026-03-08

**Authors:** Rebecca Beni, H. K. Sekhon Inderjit Singh, H. Harper, E. Lozano, A. Goede

**Affiliations:** 1 Department of Surgery, Buckinghamshire Healthcare NHS Trust, Aylesbury, HP21 8AL UK; 2 Department of Surgery and Cancer, Imperial College London, Du Cane Road, London, W12 0HS UK

**Keywords:** Rectal cancer, Watch-and-wait, LumenEye, Digital rectoscopy, Flexible sigmoidoscopy

## Abstract

**Background:**

Digital rectoscopy may reduce demand on endoscopy units, particularly as part of a watch-and-wait (WAW) organ preservation strategy for rectal cancer. LumenEye is a digital rigid rectoscope offering high-resolution rectal visualisation, video streaming and biopsy capability without sedation. This study aimed to evaluate the safety and feasibility of LumenEye in a novel outpatient colorectal clinic primarily managing WAW patients.

**Methods:**

A single centre, retrospective analysis of all patients who underwent a LumenEye examination in this clinic between August 2023 and November 2024 was conducted. Demographic, procedural, referral and outcome data were collected from electronic health records. Descriptive statistics summarised findings, while chi-squared and logistic regression analyses identified factors associated with complications or early termination.

**Results:**

A total of 327 procedures were performed; mean patient age was 67.6 years. Most procedures (54.1%, *n* = 177) were for WAW patients, with 52.6% of referrals originating from the colorectal multidisciplinary team. LumenEye was generally well tolerated: pain requiring analgesia occurred in 5.8% (*n* = 19), early termination in 2.1% (*n* = 7) and minor bleeding in 1.2% (*n* = 4). No perforations or infections occurred. One local cancer recurrence (0.3%) was missed and subsequently identified on flexible sigmoidoscopy 3 months later. Pain and bleeding were significant predictors of early termination (*p* < 0.001).

**Conclusions:**

LumenEye is a safe and practical tool for outpatient rectal examination including patients with rectal cancer undergoing WAW organ preservation strategy. Its minimal support requirements and favourable safety profile suggest that it may be a valuable alternative to traditional endoscopy, particularly in settings where access is limited.

**Supplementary Information:**

The online version contains supplementary material available at 10.1007/s10151-025-03279-0.

## Introduction

Rectal cancer is the fourth most common cancer in the UK [1]. The mainstay of treatment is resectional surgery, however, this is a major undertaking and can be significantly morbid with a decreased quality of life [2, 3]. As a result, alternative organ preservation options for early cancers have been sought which include the use of chemoradiotherapy to achieve a clinical complete response in the hopes of deferring or avoiding surgery altogether. From 6% to 28% of patients who undergo organ preservation chemoradiotherapy may achieve clinical complete response and require monitoring in a ‘watch-and-wait’ (WAW) manner [4, 5].

In cases where a WAW approach is adopted, intensive post-treatment monitoring of the rectum is required to detect early recurrence [6]. This is traditionally performed in the endoscopy unit with flexible sigmoidoscopy. However, frequent flexible sigmoidoscopy can be challenging for patients [7], and cause a significant cost to the healthcare system, with each outpatient procedure costing the NHS nearly £1000 [8]. The recently developed LumenEye device may address these challenges [9]. LumenEye is a digital rigid rectoscope with a high-definition camera, capturing images and videos of the rectal canal up to 20 cm [9, 10]. These images are displayed on a high-resolution monitor, can be shared remotely with colleagues and the device allows for biopsy collection when needed [9, 10]. Key advantages of LumenEye include its suitability for use in an outpatient clinic setting, its avoidance of sedation and shorter procedure duration [8].

**Fig. 1:**
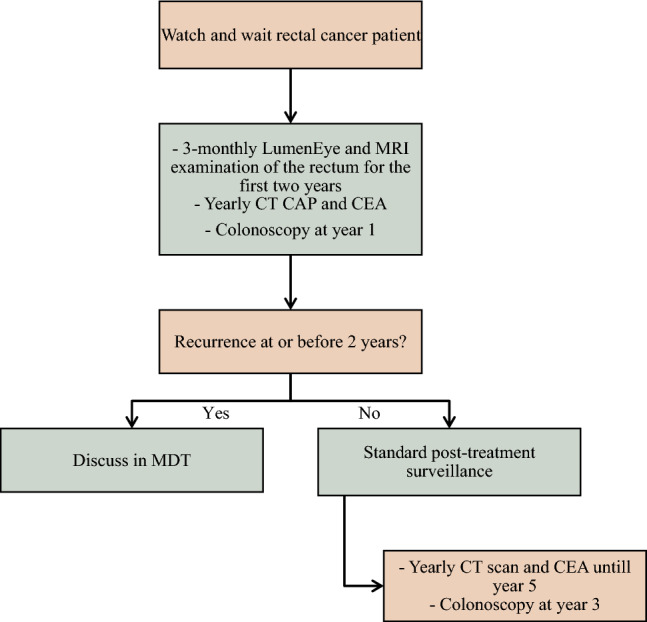
Colorectal cancer surveillance pathway at Buckinghamshire NHS Trust. MRI, magnetic resonance imaging; CT CAP, computed tomography of the chest, abdomen and pelvis; CEA, carcinoembryonic antigen

At Buckinghamshire Healthcare NHS Trust, a dedicated novel LumenEye clinic was established in August 2023 primarily to monitor patients with rectal cancer who had completed chemoradiotherapy, achieved a clinical complete response and opted for organ preservation and WAW monitoring. Prior to the LumenEye clinic, flexible sigmoidoscopy was utilised every 3 months for the first 2 years. Figure 1 shows our WAW protocol. In addition to WAW patients, the LumenEye clinic also accepts referrals for evaluation of other anorectal symptoms, particularly where urgent visualisation or biopsy is warranted.

The primary aim of this study is to evaluate the suitability of LumenEye for WAW patients with regard to missed local recurrence. Missed local recurrence was defined as any malignancy which was diagnosed on subsequent investigations during the follow-up period.

The secondary aims were to assess safety and explore patient compliance in the form of procedure termination. The secondary aims were not limited to WAW patients, that is, indication for LumenEye. Procedure termination was defined as any documentation in the clinical notes indicating that the procedure was interrupted or not completed as planned.

## Methods

This was a retrospective study performed at a single district general hospital (DGH) in England. The study is reported in accordance with the Strengthening the Reporting of Observational Studies in Epidemiology (STROBE) statement [11]. Power analysis for sample size requirement was not performed as this was an exploratory study.

### LumenEye clinic setup

Eligible WAW patients with rectal cancer are referred primarily via the colorectal cancer multidisciplinary team (CRC MDT). The LumenEye clinic runs biweekly. The procedure is performed by a senior colorectal surgeon, and is supported by a nurse or healthcare assistant, who also acts as a chaperone. Patients receive a phosphate enema as bowel preparation prior to the procedure. When local recurrence is suspected, targeted biopsies can be obtained immediately. The senior colorectal surgeon is also in charge of requesting and reviewing the magnetic resonance imaging (MRI), carcinoembryonic antigen (CEA) and computed tomography (CT) imaging for these patients in those first 2 years. A successful LumenEye procedure was defined as one performed or supervised by a healthcare professional certified in lower gastrointestinal endoscopy, in which the patient tolerated the examination and satisfactory visualisation of the rectum was achieved. In addition to WAW patients, the LumenEye clinic also accepts referrals for evaluation of other anorectal symptoms, particularly where urgent visualisation or biopsy is warranted.

### Patient selection criteria

Electronic healthcare records of all the patients attending the LumenEye clinic between August 2023 and November 2024 were screened. Inclusion criteria comprised all patients who underwent a LumenEye examination irrespective of indication. Patients were excluded if they did not undergo the procedure or if documentation was incomplete.

### Variable selection

Variables were selected on the basis of clinical relevance, and their potential association with procedure success and safety. Demographic, procedural and outcome variables were collected for all patients. All the patients were followed up for a minimum of 3 months to accurately detect readmissions for complications or detection of pathology that had been missed on LumenEye.

### Data validation

Several steps were taken to verify data accuracy and outcomes. Electronic records were cross-checked by two investigators (R.B. and H.H.) to confirm accuracy of variables. Discrepancies were resolved by discussion with a third investigator (H.K.S.I.S.). Follow-up data up to 3 months were verified against hospital admission records and subsequent endoscopic or radiologic reports to ensure accurate identification of complications and missed local recurrence. Definitions of key variables such as procedure termination and missed local recurrence were standardised before data extraction to ensure consistency.

### Data analysis

All patients undergoing the LumenEye procedure were subdivided into two cohorts on the basis of the indication for the procedure. Subgroup one consisted of all patients who underwent LumenEye for reasons other than WAW (non-WAW) and subgroup two consisted of patients undergoing LumenEye as per the WAW protocol. Data analysis was performed using IBM Statistical Package for the Social Sciences (SPSS) (version 30.0; SPSS Inc, Chicago, IL) [12]. Statistical significance was defined as a *p*-value < 0.05. Descriptive statistics was used to describe patient demographics, medical history, referral details and procedural outcomes. Demographic differences between the cohorts were assessed using independent samples *t*-tests or Mann–Whitney *U* tests for continuous variables depending on normality. Statistical significance for association in both cohorts was assessed using chi-squared tests. Variables with significant associations were further examined for causation using univariate, and if needed, multivariate logistic regression models.

## Results

### General results

A total of 327 LumenEye procedures were completed between August 2023 and November 2024; 177 procedures (54.1%) were performed for patients undergoing WAW, followed by referrals for rectal bleeding (10.7%) and change in bowel habit (10.4%) (Supplementary Fig. S1). Patients were primarily referred from the CRC MDT (52.6%). Supplementary Fig. S2 shows the remaining referral sources irrespective of indication.

The baseline demographic characteristics are presented in Table 1. The two cohorts did not differ significantly in age, sex distribution, family history of bowel cancer or operator characteristics. Mean follow-up duration was 8.3 months (range 3.1–16.7 months). Compared with the non-WAW cohort, WAW patients were more often male (*p* < 0.001), more likely to have received bowel preparation (*p* = 0.005) and less likely to be on anticoagulation (*p* < 0.001).

**Table 1:** Baseline demographic characteristics of patients undergoing LumenEye and of the watch-and-wait subgroup

	Overall cohort (*n* = 327)	Non-WAW subgroup (*n* = 150)	WAW subgroup (*n* = 177)
Age	67.64 ± 14.91 (23–92)	65.48 ± 17.21 (23–92)	69.47 ± 12.41 (33–93)
Gender			
Male	56.9%	55.3%	67.2%*
Female		44.7%	32.8%
Previous endoscopy			
No	22.9%	50.0%	0.0%*
Colonoscopy	55.4%	32.7%	74.6%
Flexible sigmoidoscopy	21.7%	17.3%	25.4%
On anticoagulation			
Yes	8.0%	14.7%	2.3%*
No	92.0%	85.3%	97.7%
Family history of bowel cancer			
Yes	0.3%	0.7%	0.0%
No	99.7%	99.3%	100.0%
Operator			
SAS surgeon	98.8%	97.3%	100.0%
Consultant surgeon	0.3%	0.7%	0.0%
Surgical trainee	0.9%	2.0%	0.0%
Assistant			
HCA/nurse	96.0%	94.7%	97.2%
Surgical trainee	2.8%	4.7%	1.7%
Medical student	0.9%	0.0%	1.1%
Consultant	0.3%	0.6%	0.0%
Bowel preparation			
Yes	74.6%	67.3%	80.8%*
No	25.5%	32.7%	19.2%

^*^Statistically significant difference between WAW and non-WAW subgroups (*p* < 0.005)

### Diagnostic findings and next steps

**Fig. 2:**
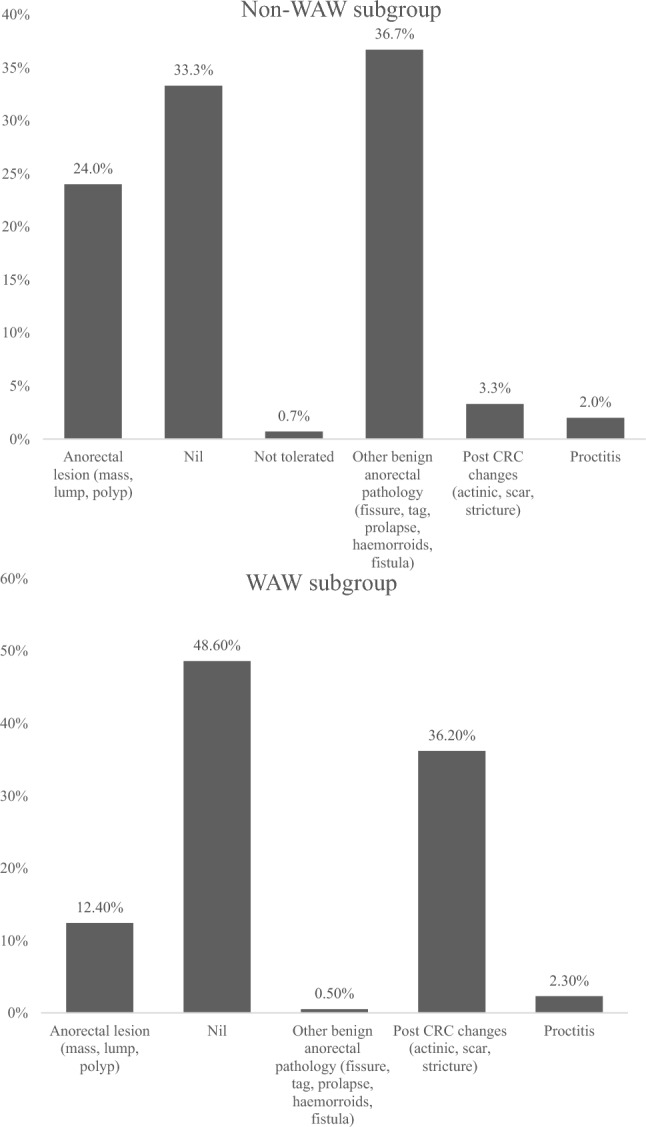
Findings on LumenEye examination split by rectal cancer surveillance indication

In the WAW subgroup most patients maintained clinical complete response with no identifiable pathology (48.6%). A further 36.2% demonstrated changes consistent with previous rectal cancer treatment, including actinic effects, scars and tattoos (Fig. 2). A total of 12.4% of patients had a lesion concerning for recurrence and 98.3% of the patients in this cohort continued on the WAW pathway (Fig. 1) following their appointment.

In the non-WAW cohort, most patients had benign rectal pathology identified (36.7%), followed by a normal procedure (33.4%); 24.0% had anorectal lesions that required further investigation (Fig. 2). A portion of these patients (3.3%) displayed post-CRC changes despite not being on WAW pathway, as these were patients with historical CRC (> 5 years) referred for other indications. In this cohort, patients were primarily discharged (28.7%) or referred for surgery for benign conditions (16.0%) (Supplementary Fig. S3).

### Safety profile

**Fig. 3:**
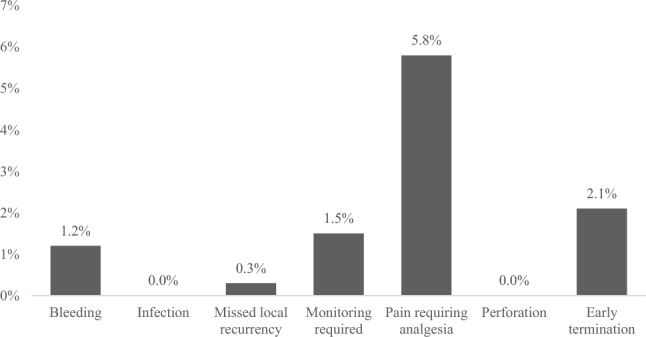
Complications rate for patients undergoing LumenEye (overall cohort)

Analysis of the overall cohort showed that the most common complication was pain requiring analgesia in 5.8% (95% CI 3.3–8.3%) of the procedures. In addition, 2.1% (95% CI 0.6–3.8%) of the procedures resulted in early termination due to pain, and 1.2% (95% CI 0.2–2.4%) resulted in bleeding. No cases of perforation or infection were reported (Fig. 3). A diagnosis of local recurrence was missed in one WAW patient. In this patient, the visualisation was optimal, with no pain. The LumenEye procedure had detected a 2 cm scar that was not friable or stenosing. The subsequent MRI organised by the specialist was equivocal (poor quality and unclear if there was recurrence or complete response) resulting in a decision by the CRC MDT to organise a flexible sigmoidoscopy in 3 months. At the time of this flexible sigmoidoscopy, the 2 cm scar now had a necrotic centre with biopsies confirming local recurrence.

### Predictors of procedure termination

Pain (*p* < 0.001) and bleeding (*p* = 0.001) were significantly associated with early procedure termination (Table 2). On univariate analysis, pain (OR 27.1, *p* < 0.001) and bleeding (OR 17.6, *p* = 0.019) were both significant predictors. This remained true in multivariate regression, where pain (OR 40.3, 95% CI 6.9–237.5, *p* < 0.001) and bleeding (OR 50.3, 95% CI 3.5–716.9, *p* = 0.004) were independent predictors (Table 3).

**Table 2:** Chi-squared test to investigate associations between missed local recurrence, bleeding, pain, completion of procedure and selected risk factors (*p*-value)

	Overall cohort (*n* = 327)				Non-WAW subgroup (*n* = 150)				WAW subgroup (*n* = 177)			
	Missed local recurrence	Bleeding	Pain	Early termination	Missed local recurrence	Bleeding	Pain	Early termination	Missed local recurrence	Bleeding	Pain	Early termination
Age older than 60 years	0.547	0.942	0.572	0.325	^a^	0.485	0.786	0.972	0.600	0.614	0.130	0.055
Gender	0.250	0.462	0.128	0.119	^a^	0.264	0.052	0.0.52	0.151	0.983	0.632	0.484
Previous endoscopy	0.585	0.272	0.445	0.582	^a^	0.316	0.117	0.096	^b^	^b^	^b^	^b^
Anticoagulation	0.768	0.554	0.187	0.432	^a^	0.677	0.153	0.300	0.879	0.791	0.660	0.879
Operator	0.994	0.975	0.883	0.957	^a^	0.986	0.850	0.918	^b^	^b^	^b^	^b^
Assistant	0.998	0.983	0.841	0.961	^a^	0.972	0.716	0.832	0.985	0.957	0.885	0.985
Bowel prep	0.559	0.986	0.322	0.051	^a^	0.485	0.692	0.356	0.625	0.531	0.158	**0.040**
Missed local recurrence	–	0.911	0.804	0.882	^a^	^a^	^a^	^a^	–	0.895	0.827	0.940
Bleeding	–	–	0.617	**0.001**	^a^	–	0.778	0.838	–	–	0.704	** < 0.001**
Pain	–	–	–	** < 0.001**	^a^	–	–	** < 0.001**	–	–	**-**	0.827

^a^Incalculable as no missed pathologies occurred in the non-WAW subgroup

^b^Incalculable as only one SAS surgeon performed WAW procedures, and all WAW patients had previous endoscopy. Bolded risk factors are independent variables

**Table 3:** Univariate logistic regression of risk factors associated with early termination

	Overall cohort (*n* = 327)	Non-WAW subgroup (*n* = 150)	WAW subgroup (*n* = 177)
Lack of bowel preparation	–	–	OR 0.996 (CI NE), *p* = NE
Bleeding	OR 17.611 (95% CI 1.6–194.7) p = 0.019*	–	OR 0.995 (CI NE), *p* = NE
Pain	OR 27.11 (95% CI 5.6–132.7) *p* < 0.001*	OR 39.1 (95% CI 6.1–251.3) *p* < 0.001	–

NE, not estimable due to sparse data/complete separation

“–” indicates no association on chi-squared, so not entered regression. “*” indicates that the data remained significant on multivariate analysis

In the non-WAW subgroup, pain was the only significant predictor of early termination (*p* < 0.001) (Table 2), and this association remained robust on univariate logistic regression (OR 39.1, 95% CI 6.1–251.3; *p* < 0.001) (Table 3). In the WAW subgroup, bleeding (*p* < 0.001) and absence of bowel preparation (*p* = 0.040) were both associated with early termination (Table 2). Due to the small number of termination events in this group, logistic regression could not reliably estimate odds ratios (Table 3).

## Discussion

This study describes the real-world use of LumenEye in an outpatient setting on primarily patients with rectal cancer undertaking WAW monitoring post-chemoradiotherapy treatment. Our study describes not only our protocol, but also that LumenEye is safe in this cohort of patients. To our knowledge, this is one of the largest reported series, encompassing a 15-month period of clinical use.

Safety, efficiency, patient compliance and cost-effectiveness are usually the three pre-requisites for the successful uptake of any new technology in the medical field, especially in the National Health Service (NHS). The protocol describes a self-sufficient clinic requiring a senior colorectal clinician and a nurse or HCA as an assistant. These are readily available in the majority of colorectal clinics in the United Kingdom. This is in contrast with the larger infrastructure and team required for flexible sigmoidoscopy. The added benefit of this pathway for WAW patients is that the optical evaluation of the rectum for recurrence is performed by the same senior surgeon who reviews the imaging evaluation of the rectum allowing for better concordance between the findings and improved continuity of care for the patient.

The safety profile of LumenEye is reassuring. We did not report any significant adverse events, which has also been shown in a smaller retrospective study of 110 patients undergoing LumenEye for lower gastrointestinal symptoms in primary care [9]. We have reported a missed local recurrence rate of 0.3% (1 patient). Whether this represents a true missed recurrence is open to debate, as mucosal visualisation was documented as satisfactory at the time of the LumenEye examination. It is unlikely that the use of flexible sigmoidoscopy in place of LumenEye would have altered subsequent clinical management, which consisted of continued radiological surveillance with MRI followed by repeat procedure (flexible sigmoidoscopy or LumenEye) to visualise the rectal mucosa. Nevertheless, this case was conservatively classified as a missed lesion to avoid underestimation of risk. Even with this conservative approach, the observed rate of missed pathology remains acceptably low and is substantially lower than the 2–6% rate of missed colorectal cancers reported in the lower gastrointestinal endoscopy literature [13].

While 5.8% experienced pain, only 2.1% overall required termination despite analgesia. Options for analgesia trialled in our cohort included topical lidocaine or EMLA. In comparison, rates of up to 15–25% for pain and discomfort have been reported with flexible sigmoidoscopy [14]. These rates vary with the use of sedation. Our subgroup analyses highlight areas for further exploration. In the overall cohort, bleeding and pain were associated with early termination and remained an independent predictor on multivariable logistic regression, with a similar association noted with pain in the non-WAW subgroup. Strategies to further optimise comfort may help reduce these instances. If upcoming patient satisfaction and cost analyses from our unit support the usability and economic value of LumenEye, these findings may help inform decisions about its role within NHS colorectal pathways. Such information could be particularly relevant for DGH with limited endoscopy capacity and increasing rectal cancer surveillance demands.

Our study does have several limitations. Firstly, the retrospective nature of the study introduces risks of information bias and incomplete documentation. Secondly, most procedures were performed by a single experienced surgeon in a single centre, and results may differ with less experienced users or in different populations, reducing generalisability. We also make no measure of interobserver variability between LumenEye users. Thirdly, LumenEye is limited to the rectosigmoid and cannot identify more proximal pathology which might be visualised with flexible sigmoidoscopy.

An international prospective multicentre cohort study is currently underway to evaluate the rate of rectal cancer response with LumenEye. They will also assess inter-observer variability [15]. Regression analyses were underpowered due to low event numbers, leading to wide confidence intervals and limiting the reliability of identified associations. These findings should therefore be interpreted as exploratory and hypothesis generating.

## Conclusions

This study provides the first real-world data on the use of the LumenEye rectoscope in an outpatient setting within a single DGH. Over a 15-month period, LumenEye proved to be a safe and feasible tool for rectal cancer patients undergoing WAW and for other lower gastrointestinal indications. With a very low complication rate, LumenEye offers an alternative to traditional flexible sigmoidoscopy.

## Supplementary Information

Below is the link to the electronic supplementary material.

Supplementary file1 (DOCX 74 KB)

## Data Availability

The datasets used and analysed during the current study are available from the corresponding author on reasonable request.

## Declarations

### Permission to reproduce material from other sources

None. Not applicable.

### Clinical trial registration

None. Not applicable.
