# Supplementary material for: Evaluating the clinical use and safety of LumenEye in an outpatient colorectal clinic: a single-centre retrospective cohort study
Source: Tech Coloproctol. 2026 Mar 8;30(1):42. doi: 10.1007/s10151-025-03279-0 (PMC12979350; doi:10.1007/s10151-025-03279-0)
Supplement: Supplementary file 1 — Supplementary file1 (DOCX 74 KB) [file 10151_2025_3279_MOESM1_ESM.docx]

**Supplementary materials**

*Supplementary figure* 1 – Origin of referral to LumenEye clinic (Overall cohort)

*Supplementary figure* 2 - Reason for referral to LumenEye clinic (Overall cohort)

*Supplementary figure* 3 – Next steps for patients seen in LumenEye clinic
